# Supplementary material for: The Construction and Exploration of a Comprehensive MicroRNA Centered Regulatory Network in Foxtail Millet (Setaria italica L.)
Source: Front Plant Sci. 2022 May 6;13:848474. doi: 10.3389/fpls.2022.848474 (PMC9121102; doi:10.3389/fpls.2022.848474)
Supplement: Supplementary file 3 [file Data_Sheet_3.zip › Sit-miR393b_Seita.7G233200.1_71_TPlot.pdf]

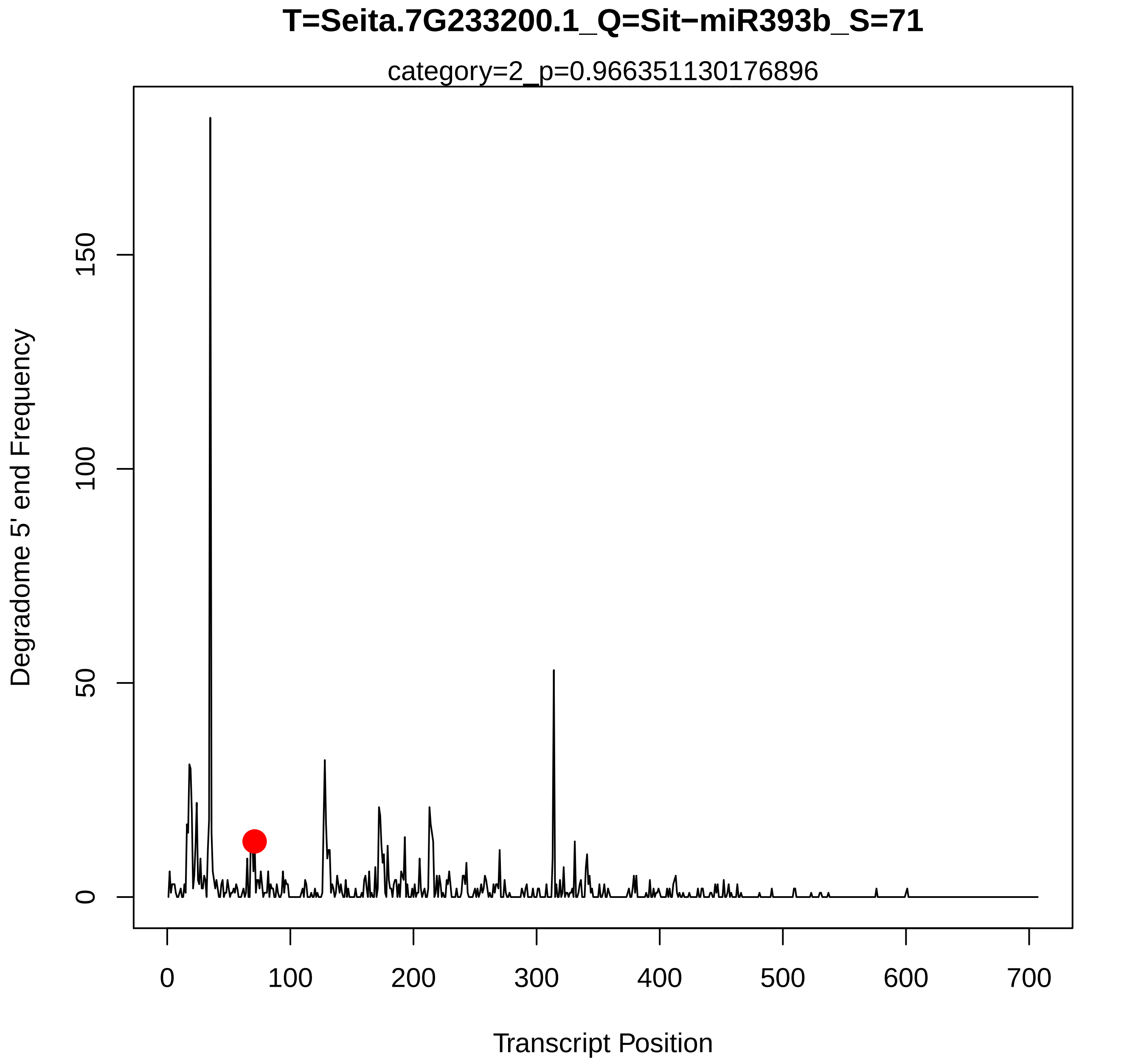

T=Seita.7G233200.1_Q=Sit−miR393b_S=71
category=2_p=0.966351130176896
Degradome 5' end Frequency
0
50
100
150
0
100
200
300
400
500
600
700
Transcript Position
